# The Effect of Platelet-Rich Fibrin, Calcium Sulfate Hemihydrate, Platelet-Rich Plasma and Resorbable Collagen on Soft Tissue Closure of Extraction Sites

**DOI:** 10.3390/jfb8020017

**Published:** 2017-05-25

**Authors:** Lisa M. Yerke, Amal Jamjoom, Talal M. Zahid, Robert E. Cohen

**Affiliations:** 1 Department of Periodontics and Endodontics, School of Dental Medicine, University at Buffalo, State University of New York, 250 Squire Hall, Buffalo, NY 14214, USA; amal.g.jamjoom@gmail.com (A.J.); rcohen@buffalo.edu (R.E.C.); 2 Department of Periodontology, King Abdulaziz University, University District, Jeddah 22252, Saudi Arabia; tzahid@kau.edu.sa

**Keywords:** extraction socket, platelet-rich protein, platelet-rich fibrin, calcium sulfate, soft tissue healing

## Abstract

Rapid and complete soft tissue healing after tooth extraction minimizes surgical complications and facilitates subsequent implant placement. We used four treatment methods and assessed changes in soft tissue socket closure following tooth extraction in humans. The effects of platelet-rich fibrin-calcium sulfate hemihydrate (PRF-CSH), platelet-rich plasma-calcium sulfate hemihydrate (PRP-CSH), a resorbable collagen dressing (RCD), and no grafting material were compared in a randomized, controlled pilot study with a blinded parallel design (*N* = 23). Patients with a hopeless tooth scheduled for extraction were randomly assigned to one of the four treatment groups. Socket measurements were obtained immediately after extraction and treatment, as well as after 21 days. There was a significant decrease in the total epithelialized external surface area of the extraction sockets in each group at all time points. However, there were no significant differences in soft tissue closure (*p* > 0.05) at any time point and PRF-CSH or PRP-CSH did not provide any additional benefit to enhance the soft tissue closure of extraction sockets compared with either RCD or sites without graft.

## 1. Introduction

Tooth extraction leads to vertical and horizontal ridge resorption that can make implant placement difficult or impossible [1]. However, it is well established that post-extraction ridge preservation can be beneficial prior to implant placement [1,2]. Although some studies have shown that ridge preservation does not completely prevent bone loss post-extraction, such procedures aid in reducing the extent of that loss [3].

Ridge preservation can be performed using a variety of materials. Ideally, graft materials provide osteogenic, osteoinductive, and/or osteoconductive properties, and also provide mechanical support and supply a framework for osteogenic cells to stimulate bone production [4]. In addition, rapid socket closure and re-epithelialization may assist in graft retention and exclusion of debris, as well as improve patient comfort.

A variety of graft materials are available for ridge preservation within extraction sockets. Autogenous bone can be transferred from one position to another within the same individual. Allogenic bone grafts (allografts) are transferred from one individual to another in the same species. They can be fresh-frozen or freeze-dried, and mineralized or demineralized. Xenografts are derived from other species and are derived from a variety of animals including bovine, porcine, and equine sources. Alloplasts are synthetic bone substitutes that act as a biologic filler, such as medical grade calcium sulfate hemihydrate (CSH) or hydroxyapatite. When CSH is implanted, it dissolves into calcium and sulfate ions. Calcium ions subsequently combine with phosphate ions from the host to form calcium phosphate, which provides a scaffold for bone ingrowth into the defect. In addition, CSH has angiogenic and hemostatic properties [5].

The incorporation of growth factors during regenerative therapy provides the potential to accelerate new bone formation and enhance ridge preservation [4]. Examples include platelet-rich plasma (PRP), platelet-rich fibrin (PRF), bone morphogenic proteins, and enamel matrix proteins. PRP is obtained by mixing blood and utilizing a two-stage centrifugation protocol that isolates the platelet concentrate, which contains 6–8 times the amount of growth factors compared to whole blood [6]. However, the use of an anticoagulant to obtain PRP is disadvantageous to wound healing. PRF potentially enhances wound healing because it does not contain an anticoagulant and, in addition, it contains more white blood cells than PRP, leading to higher numbers of white blood cells, which translates to having an increased number of macrophages that are responsible for releasing growth factors such as transforming growth factor beta, platelet-derived growth factor, and vascular endothelial growth factor [6]. Those cells and cytokines are critical to wound healing. PRF has been reported to maintain and stabilize grafts, integrate into regenerative sites, facilitate cellular migration, and enhance soft tissue healing [7,8].

We wished to determine if factors potentially influencing bone regeneration and preservation could be applied to the overlying soft tissues of extraction sockets. Suttapreyarsi and Leepong found that recent extraction sockets given PRF had accelerated soft tissue healing compared to control at four weeks [9]. Dutta et al. observed that extraction sockets receiving PRP and PRF were associated with less pain, swelling, and faster soft tissue healing compared to hydroxyapatite at the third, seventh and fourteenth day [10]. However, we could not find any studies comparing PRP and PRF over a three-week period in potential future implant non-third molar implant sites. Consequently, the aim of this pilot study was to compare the soft tissue closure of human tooth extraction sites by measuring the extent of socket epithelialization immediately grafted with platelet-rich fibrin-calcium sulfate hemihydrate (PRF-CSH), platelet-rich plasma-calcium sulfate hemihydrate (PRP-CSH), resorbable collagen dressings (RCD), and at sites with no graft placement, after three weeks.

## 2. Results

The demographic characteristics of the study population are listed in Table 1. The population included 12 females and 11 males with a mean age of 61 years (40–72 years). There were no statistically significant differences (*p* > 0.05) with respect to mean age or gender at baseline, prior to the extraction procedures.

All patients completed the 21-day follow-up examination; there were no dropouts. Clinical healing was uneventful and similar in all groups. No signs of infection were noted with any patient, nor were there any unusual clinical outcomes. The majority of grafted sites achieved complete epithelial closure after 21 days in all groups. A decrease in the total epithelialized external surface area of the extraction sockets was noted at 10 days and 21 days following tooth extraction. Analysis of variance revealed no significant differences among any of the groups at 21 days (Table 2), implying that soft tissue healing was independent of the grafting methods used in this study.

## 3. Discussion

The purpose of this study was to compare the soft tissue healing using three different grafting materials (PRP-CSH, PRF-CSH, and RCD) and a non-grafted control at 21 days post-extraction. All groups were healing without complication at 21 days. This result is consistent with wound healing post-extraction when no graft is placed. Immediately following extraction, a blood clot consisting of erythrocytes and platelets in a fibrous matrix is formed within the socket. Granulation tissue, a highly vascularized new connective tissue, then starts to form at 48 h and is complete by day 7. The granulation tissue is replaced by connective tissue in approximately 30 days. Re-epithelialization concurrently begins at four days post-extraction and is completed by six weeks, depending on the tooth site [11]. Our results confirmed soft tissue healing at three weeks, regardless of the extraction site being located in the anterior or posterior region.

Intini [12] and Kutkut [13] showed that CSH possesses angiogenic and hemostatic properties, and is also biocompatible, biodegradable, safe, and non-toxic. Similarly, we did not note any CSH complications when used as a grafting material for extraction socket preservation, and soft tissue coverage at extraction sites occurred uneventfully. Our results are similar to those of a previous study investigating the effects of calcium sulfate placed at extraction sites that reported soft tissue closure between 21 to 28 days at both grafted and non-grafted sites [14]. Similarly, our results showed that neither PRP-CSH nor PRF-CSH resulted in significantly faster soft tissue healing compared to resorbable collagen dressing or sites without any grafted material.

PRP and PRF appear to have many potential applications in periodontal and dento-alveolar surgery. Nevertheless, reports describing their efficacy are mixed. In a histological and radiographic study, Cheah et al. showed that PRP-CSH had a significantly beneficial effect on ridge preservation and augmentation with regard to horizontal and vertical bone resorption, and also appeared to increase the percentage of newly formed bone [15]. Sammartino et al. conducted a split mouth histological study analyzing the effect of incorporating PRP onto resorbable collagen membranes at impacted third molar extraction sites [16]. They showed that the composite PRP-collagen membrane demonstrated earlier bone maturation (but not increased bone regeneration). Kutkut et al. showed that adding PRP to both CSH and resorbable collagen membrane had a positive effect on both bone maturation and regeneration at extraction sockets [13]. In our study, PRP did not exhibit any clinically beneficial effect when added to CSH grafting material.

Del Corso et al. suggested that the use of PRP gels has declined due to their expense and preparation time [17]. Relative to PRP, PRF appears to be more user-friendly, easier to prepare, and less expensive. Consequently, PRF is being used with increasing frequency in periodontal and oral surgery compared to PRP. Jain et al. suggested that PRF supports the three primary aspects of wound healing—angiogenesis, immunity, and epithelial proliferation—thus supporting its use for wound protection and acceleration of healing [18].

Compared to PRP, PRF is reported to result in improved tissue healing and bone formation. In an in vitro study, Dohan and Choukroun [19] found that growth factors are gradually released from PRF, which provides a more pronounced effect on cellular proliferation and differentiation compared to PRP, which releases those factors over a shorter time period [20]. Yelamali and Saikrishna similarly observed improved soft tissue healing at one week in third molar extraction sites when PRF was used compared to when PRP was used [21]. They also concluded that the improved soft tissue healing associated with PRF was partially attributed to its ability to release growth factors in a controlled manner over a longer period of time. However, since no hematological analyses were performed on the blood samples used for PRP and PRF preparations in our study, the growth factor concentration may have varied between the patients and study groups.

Limitations of our study include site measurement via a periodontal probe, short observation time, and lack of histology. All tooth types from both arches were used, which might have introduced more variability. In addition, a larger sample size might have enabled differences in study groups to become significant. 

Future studies can individually explore the relationship of CSH and RCD on epithelialization, with or without the addition of PRP or PRF.

## 4. Materials and Methods 

This study was conducted in accordance with the Helsinki Declaration of 1975 (revised in 2000). The study protocol was reviewed and approved by the Health Science Institutional Review Board of the University at Buffalo, State University of New York (Project #P/E0821111A).

### 4.1. Study Design

**Figure 1:**
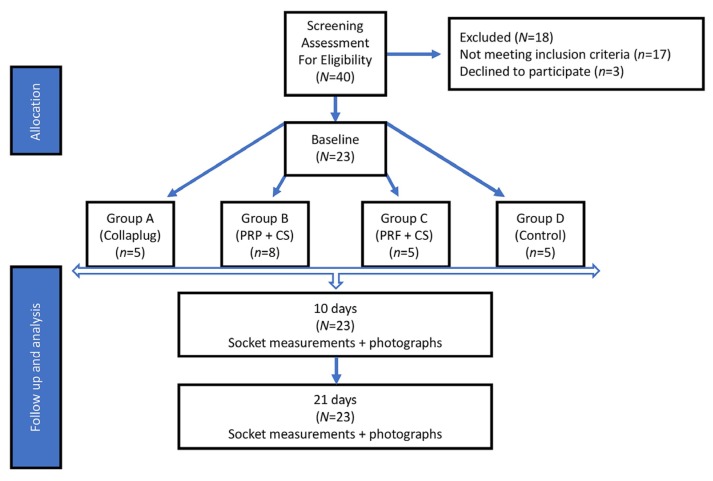
Diagram of subject allocation, the randomization of subjects into the four study groups, and the observation time points. PRP = platelet-rich plasma; PRF = platelet-rich fibrin; CS = calcium sulfate hemihydrate.

This study was a single site, randomized, single masked, controlled clinical trial. The primary outcome variable was the soft tissue closure of extraction sites. At the screening visit, the patients’ medical history was reviewed and a dental assessment, including periodontal charting, bleeding on probing, and the O’Leary plaque index [22], was performed. Oral hygiene instructions were provided and scaling and root planing was performed within two weeks of the screening visit. At the baseline visit, subjects were randomly assigned to one of the treatment groups (Figure 1). 

### 4.2. Study Population

Twenty-three healthy subjects requiring extraction of at least one tooth (11 males and 12 females) were recruited from the University at Buffalo, School of Dental Medicine patient care clinics. Criteria for exclusion were medications associated with gingival overgrowth (e.g., cyclosporine, Dilantin, calcium channel blockers), current use of non-steroidal anti-inflammatory medications, medications that may affect bone healing (e.g., bisphosphonates), the presence of systemic or local infection, the presence of systemic diseases (including thyroid disease, diabetes, and kidney or liver disease), pregnant or nursing women, smoking, and inability to adhere to the study schedule. The indication for extraction included crown or root fractures, non-restorable caries, poor crown-to-root ratio, supra-erupted teeth with no opposing occlusion, or occlusal interferences in a patient with partial dentures. To be included in the study, alveolar sockets were required to have three or four bony walls. Eligible subjects subsequently were assigned to four groups by a computer-generated random number table. The computer-generated number table and the subject assignments was completed by an author other than the author who was responsible for the subject examinations.

### 4.3. Extraction Procedures

Prior to tooth extraction, 5 mL of blood was drawn from each subject’s median cubital vein and collected into tubes containing 1.5 mL of 10% trisodium citrate (Vacutainer^®^ tubes, Becton Dickinson, Franklin Lakes, NJ, USA) for Group B, or empty tubes for the three remaining groups. For patients in Groups B and C, the blood was centrifuged for 10–12 min at 400× *g*. Samples from Groups A and D were not used in the study. 

After the administration of local anesthesia (Xylocaine^®^, 2% injection with 1:100,000 epinephrine; DENTSPLY Pharmaceutical, York, PA, USA), a sulcular incision was performed with a 15C blade, and a periotome was used to separate the supracrestal periodontal ligament fibers without flap reflection. The tooth was luxated using a straight elevator and extracted with forceps in an effort to minimize stress on the buccal bone. The extraction socket was debrided of granulation tissue.

In all groups, the buccolingual and mesiodistal dimensions at the osseous crest of the extraction socket were measured at the socket midpoints immediately after extraction with a University of Michigan O-probe with Williams markings after 21 days. Extraction sites assigned to each group were treated as described below:

1. Group A: Five extraction sites were treated with RCD alone (Collaplug^®^; Zimmer Dental, Carlsbad, CA, USA) and served as a positive control. The RCD was placed in the socket apical to the free gingival margin and secured in place by a 4-0 silk horizontal mattress suture.
2. Group B: Eight extraction sites treated with 1 g CSH (DentoGen^®^, Orthogen Corporation, Springfield, NJ, USA) mixed with 240 µL of the subject’s PRP. The viscous mix was placed in the apical two-thirds of the socket and covered with a 15 mm × 20 mm resorbable collagen membrane (OraMem^®^, Salvin, Charlotte, NC, USA) that was shaped to cover the extraction site [12]. The membrane was used to cover the mixture and protect it from oral debris. It was secured in place under the free gingival margin with a silk 4-0 horizontal mattress suture.
3. Group C: Five extraction sites were treated with 1 g CSH mixed with a PRF membrane that was cut into small pieces (approximately 2 mm × 2 mm) and applied into the socket [20]. The materials were then covered with another PRF membrane that was adapted to cover the extraction site and placed just apical to the gingival margin, then secured with a silk 4-0 horizontal mattress suture.
4. Group D: Five extraction sites did not receive any grafting material after extraction (Group D) and served as a negative control. A horizontal mattress suture using 4-0 silk was used to close the site and readapt the tissues.

Following extraction, the patients received post-operative instructions and were asked to cleanse the extraction sites gently with an oral swab (Toothette Oral Swabs^®^, Sage Products, Cary, IL, USA) saturated with 0.12% chlorhexidine gluconate (PerioGard^®^, Colgate Oral Pharmaceuticals, New York, NY, USA) four times daily for two weeks. In addition, amoxicillin (500 mg three times daily for seven days) was prescribed. The patients that were allergic to penicillin were prescribed clindamycin (150 mg four times daily for seven days).

The area required for soft tissue healing was calculated by multiplying the buccolingual and mesiodistal measurements taken with the periodontal probe at each patient appointment.

Differences between groups were measured using one-way analysis of variance, and were corrected for multiple comparisons.

## 5. Conclusions

The use of PRF-CSH or PRP-CSH did not provide any additional benefit to enhance the soft tissue closure of extraction sockets compared with either RCD or with non-grafted sites. Sites from all groups healed uneventfully within the 21-day time period used in this study. Future studies using greater numbers of extraction sites, longer time periods, and histologic analysis can be considered to determine if a variety of graft materials might influence soft tissue healing, and if those changes have any influence on bone regeneration or ridge preservation.

## Figures and Tables

**Table 1:** Patient data.

Patient	Age	Gender	Tooth Extracted	Grafting Material	Bony Walls after Extraction
1	63	Male	30	Control	4
2	68	Male	2	Control	3
3	59	Female	4	Control	4
4	70	Male	18	Control	3
5	54	Male	20	Control	4
6	68	Male	5	Collagen Dressing	4
7	70	Male	15	Collagen Dressing	4
8	62	Female	15	Collagen Dressing	3
9	63	Female	15	Collagen Dressing	4
10	60	Female	31	Collagen Dressing	4
11	70	Male	2	PRP-CSH	4
12	60	Female	5	PRP-CSH	4
13	66	Female	4	PRP-CSH	4
14	65	Female	7	PRP-CSH	4
15	68	Male	15	PRP-CSH	3
16	72	Male	14	PRP-CSH	3
17	67	Male	15	PRP-CSH	3
18	40	Female	6	PRP-CSH	4
19	46	Female	4	PRF-CSH	4
20	42	Female	4	PRF-CSH	4
21	61	Male	2	PRF-CSH	3
22	56	Female	8	PRF-CSH	4
23	67	Female	29	PRF-CSH	4

PRP-CSH: Platelet-rich plasma-calcium sulfate hemihydrate; PRF-CSH: Platelet-rich fibrin-calcium sulfate hemihydrate.

**Table 2:** Soft tissue closure of extraction sites; analysis of variance among groups after 21 days.

	Sum of Squares	Df	Mean Square	F	*p*-Value
Between Groups	853.613	3	284.538	0.591	0.628
Within Groups	9142.360	19	481.177		
Total	9995.973	22			

Df: degrees of freedom; F: *F*-test statistic.
